# Anti-Inflammatory Effect of *Meriania hexamera* Sprague by Targeting Syk Kinase in NF-κB Signaling

**DOI:** 10.3390/plants12173044

**Published:** 2023-08-24

**Authors:** Ki Woong Kwon, Won Young Jang, Ji Won Kim, Jin Kyoung Noh, Dong-Keun Yi, Jae Youl Cho

**Affiliations:** 1 Department of Integrative Biotechnology, Sungkyunkwan University, Suwon 16419, Republic of Korea; nexus0322@naver.com (K.W.K.); wybest0327@naver.com (W.Y.J.); lauryun@naver.com (J.W.K.); 2 Instituto de BioEconomia, El Batan, Quito 170135, Ecuador; 3 International Biological Material Research Center, Korea Research Institute of Bioscience and Biotechnology, Daejeon 34141, Republic of Korea; lydian78@kribb.re.kr

**Keywords:** inflammation, *Meriania hexamera* Sprague, NF-κB, Syk, gastritis

## Abstract

Inflammation is a protective mechanism against harmful stimuli. There are two types of inflammation, acute and chronic, and severe diseases such as cardiovascular disease and cancer can be caused by chronic inflammation. Therefore, this research was conducted to discover new anti-inflammatory drugs. *Meriania hexamera* Sprague is a common herb in the Amazon region in South America. It is used as a traditional medical herb by natives, but no studies to date have investigated its anti-inflammatory activity. Using lipopolysaccharide (LPS), pam3CSK4 (Pam3), and poly(I:C), we studied the *M. hexamera* Sprague–Methanol Extract’s (Mh-ME) in vitro anti-inflammatory functions. Using RAW264.7 cells, we detected the released nitric oxide (NO) and mRNA expression extent of inducible nitric oxide synthase (iNOS) with pro-inflammatory proteins like tumor necrosis factor-alpha (TNF-α), interleukin-6 (IL-6), and iterleukin-1 beta (IL-1β). It was found that Mh-ME suppressed the inflammatory activities in a dose-dependent manner. In the luciferase assay, the nuclear factor kappa light chain enhancer of the activated B cells (NF-κB) pathway was inhibited by Mh-ME. Mh-ME especially acted as an inhibitor of Syk kinase according to the results from CETSA. We also confirmed that Mh-ME mitigates acute gastritis derived from HCl/EtOH in ICR mice, ameliorating the expression of IL-1β and tumor necrosis factor-alpha (TNF-α). In conclusion, Mh-ME is an herb with anti-inflammatory effects that targets Syk in the NF-κB pathway, suggesting that Mh-ME could be used as an anti-inflammatory herbal medicine.

## 1. Introduction

Inflammation is one of the most representative defense mechanisms to protect the body from stimuli including pathogens, chemical agents, and autoimmune responses [1]. Inflammation is induced by innate immune response involving macrophages, neutrophils, and dendritic cells and then eliminates the foreign pathogens. However, inflammation can also cause redness, heat, swelling, pain, and even loss of cell function [2]. In addition, there are two types of inflammation, acute and chronic; chronic inflammation can cause severe diseases such as autoimmune disease, cardiovascular disease, gastritis, pneumonia, and even cancer. Therefore, suppressing the excessive inflammatory response is important for body health.

Innate immunity occurs by mediating lymphocytes such as macrophages and neutrophils. Diversiform lymphocytes recognize pathogen-associated molecular patterns (PAMPs) of pathogens using pattern recognition receptors (PRRs) [3]. Among the PRRs, the toll-like receptors’ (TLRs) family members, which are positioned at the cell surface or in the cytoplasm, recognize lipid or protein ligands of pathogens [4]. For instance, TLR4 recognizes lipopolysaccharide (LPS), a representative pathogen in gram-negative bacteria, and initiates inflammatory signal cascades through enhancing the subsequent signals mediated by proteins, including TIR-domain-containing adapter-inducing interferon-β (TRIF) and myeloid differentiation response gene 88 (MyD88) [5,6]. After immune cells recognize the PAMPs, they activate the inflammatory signaling pathway. In the case of non-pathogenic inflammatory responses, severe damage to cells (which may be derived from physical injury or contact with toxic chemicals) causes either necrosis or apoptosis of cells, causing the release of damage-associated molecular patterns (DAMPs). Since PRRs also interact with DAMPs, non-pathogenic damages may also initiate TLR-dependent inflammatory cascades. During the inflammatory responses, macrophages activate nuclear factor-kappa B (NF-κB) or activated protein-1 (AP-1) pathways, involving phosphorylation of Src, spleen tyrosine kinase (Syk), protein kinase B (PKB, also known as AKT), and inhibitor of kappa B alpha (IκBα) [7,8]. Stimulation of the TLR4 pathway increases various inflammatory cytokines or enzymes, for example, inducible nitric oxide synthase (iNOS), interleukin-1β (IL-1β), IL-6, tumor necrosis factor-alpha (TNF-α), and cyclooxygenase-2 (COX-2) [9], leading to inflammation. In inflammatory responses for body homeostasis, immune response is important, but so is immune tolerance that inhibits excessive immune response [10]. Uncontrolled inflammation develops into chronic inflammation, which disrupts the immune tolerance and causes a variety of severe diseases, including cardiovascular disease, autoimmune disease, cancer, and inflammatory diseases such as gastritis [2,11].

*Meriania hexamera* Sprague is native to South America in Colombia and Ecuador and belongs to the Melastomataceae family. Plants belonging to the Melastomataceae family are known to have a high number of phenolic compounds, including flavonoids, terpenoids, quinones, lignans, and tannins [12]. These ingredients have anti-inflammatory, antioxidant, and antibacterial activities, helping to regenerate gastric mucosa and heal gastric ulcers. To be specific, *Meriania hernanondoi* and *Meriania nobilis* contain hydrolytic tannins with radical scavenging activities [13]. As a result, leaves of plants in the Melastomataceae family have been used as a traditional therapy. Nevertheless, there have been no previous studies that revealed the molecular mechanism of the anti-inflammatory effects of this plant and therapeutic effect on gastritis. Thus, in this study, we clarified the anti-inflammatory effects of *M. hexamera* Sprague–Methanol Extract (Mh-ME) and its associated inhibitory mechanism. Furthermore, the in vitro anti-inflammatory activities of this extract were confirmed in an in vivo model with acute gastritis, a representative inflammatory disease.

## 2. Results

### 2.1. Mh-ME Ameliorated NO Production without Inducing Death of RAW264.7 Cells

**Figure 1:**
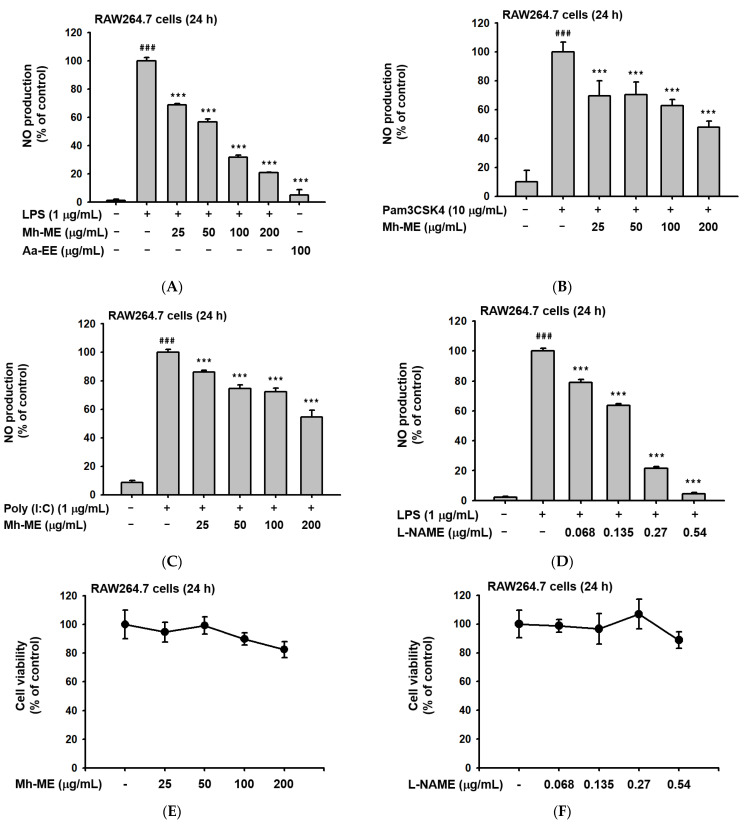
In vitro anti-inflammatory efficacies of Mh-ME. (**A**) NO synthesis intensity in RAW264.7 cells that were pretreated with Mh-ME (0–200 μg/mL) or Aa-EE (100 μg/mL) during LPS (1 μg/mL) exposure for 24 h. NO was determined by Griess assay. (**B**,**C**) NO production levels in RAW264.7 cells induced by Pam3CSK (10 μg/mL) and poly(I:C) (200 μg/mL) for 24 h. (**D**) The production of NO reduced by L-NAME (0.25–2 µM). (**E**,**F**) Viability of RAW264.7 cells treated with Mh-ME (0–200 μg/mL) and L-NAME (0–54 μg/mL) investigated using the MTT assay. ### *p* < 0.001 compared with the normal group; *** *p* < 0.001 compared with the LPS-treated group.

For reconfirmation of anti-inflammatory effects of Mh-ME in vitro, we evaluated the levels of NO in RAW264.7 cells after treating Mh-ME (0–200 μg/mL). We respectively treated LPS (derived from Gram (-) bacteria), which is a ligand for TLR4 (Figure 1A); Pam3CSK (synthetic analog of components of gram (+) bacteria), a ligand for TLR1/2 (Figure 1B); and a poly(I:C) (synthetic analog of viral nucleotides), a ligand for TLR3 (Figure 1C), in Mh-ME-treated RAW264.7 cells. We additionally used Artemisia asiatica ethanol extract (Aa-EE) as a positive control plant extract, which is known for its anti-inflammatory effect [14]. As a result, RAW264.7 cells treated with different types of pro-inflammatory inducers all showed significant reduction in NO production in a dose-dependent manner after Mh-ME treatment. Aa-EE showed 95% inhibitory activity under the same conditions (Figure 1A). In particular, it was confirmed that 200 μg/mL of Mh-ME lowered NO production to about 50% under all induction conditions. The effect was comparable to L-NAME, a well-known NO synthase inhibitor targeting hypotension (Figure 1D). Therefore, we set 200 μg/μL of Mh-ME as the target concentration and tested the cytotoxicity of the maximum 200 μg/μL of Mh-ME in RAW264.7 cells (Figure 1E,F) with an MTT assay. An amount of 200 μg/mL of Mh-ME did not lower the viability of the RAW264.7 cells.

### 2.2. Analysis of the Components of Mh-ME

**Figure 2:**
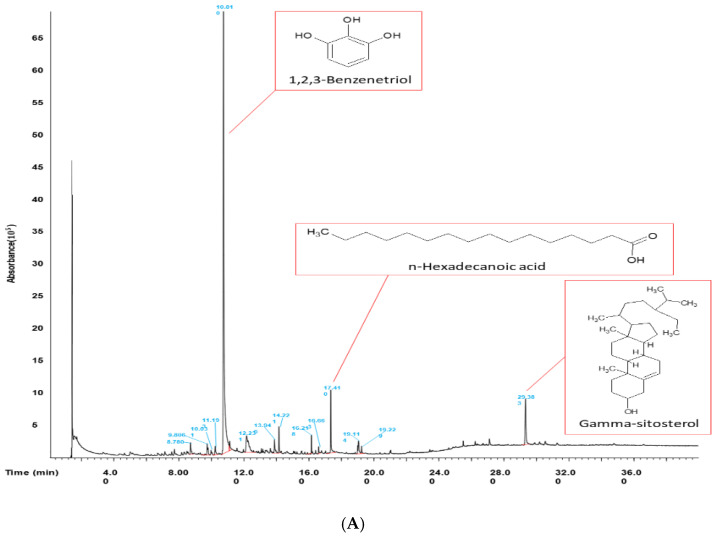

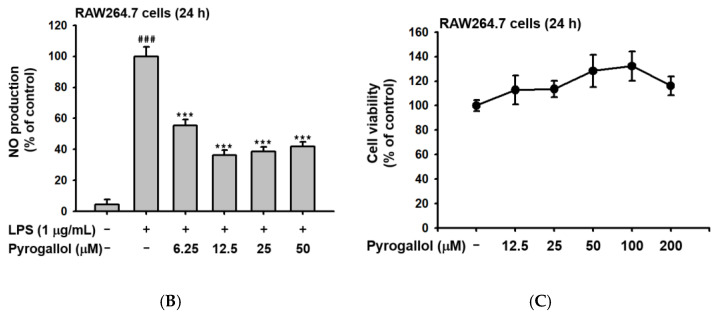
The phytochemical analysis of Mh-ME and NO-inhibitory activity of pyrogallol. (**A**) The GC-MS chromatogram of Mh-ME. (**B**) NO inhibitory activity of 1,2,3-benezetriol (pyrogallol) was examined using LPS-treated RAW264.7 cells by measuring NO production level by Griess assay. (**C**) Effect of pyrogallol on the viability of RAW264.7 cells was evaluated by MTT assay. ### *p* < 0.001 compared with the normal group; *** *p* < 0.001 compared with the LPS-treated group.

We hypothesized that the anti-inflammatory activity of Mh-ME derives from its active compounds, which may be various types of polyphenols. We examined the anti-inflammatory components of Mh-ME using gas chromatography–mass spectrometry (GC-MS). As expected, the major component contained in Mh-ME is one of the phenolic compounds, 1,2,3-benzentriol, also known as pyrogallol (Figure 2A and Table 1). As pyrogallol is the substance that Mh-ME contains the most, MTT and NO assays were additionally conducted to confirm the anti-inflammatory efficacy of pyrogallol. We confirmed that the NO production accelerated by LPS was reduced by about half after treating only 6.25 μM of pyrogallol, although there was no dose-dependent NO inhibitory pattern at higher concentrations (25 and 50 μM) (Figure 2B). Moreover, pyrogallol did not show cytotoxicity on RAW264.7 cells, and, rather, higher doses (25 to 200 μM) enhanced the viability levels of the cells (Figure 2C). Though additional work should be necessary, enhanced levels of cell viability by pyrogallol (Figure 2C) seem to affect its NO inhibitory activity, showing no dose-responsiveness. Nonetheless, this might indicate that the active compound of Mh-ME on anti-inflammatory efficacy may be pyrogallol. Since pyrogallol moiety is included in various polyphenols such as flavonoids, we additionally measured the content of total phenolic compound and quercetin in the Mh-ME (Table 2). The specific flavonoid contents in Mh-ME were further characterized via LC-qTOF-MS/MS (see Table S1). The results showed that a total of 121 phenolic compounds were detected in Mh-ME, of which some were formally reported to have strong anti-inflammatory properties (Table 3) [15,16,17].

### 2.3. Effects of Mh-ME on the Transcriptional Activation of Pro-Inflammatory Proteins

**Figure 3:**
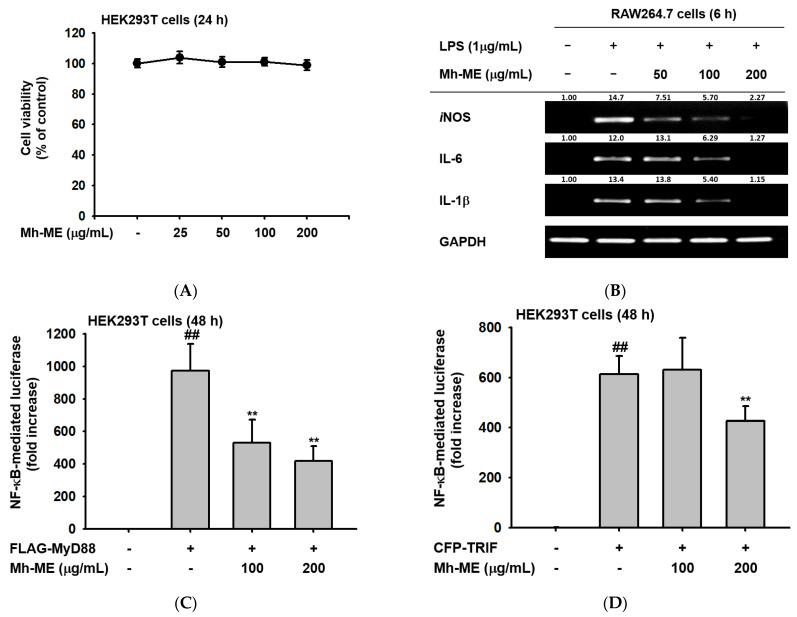
Suppressive effects of Mh-ME on the mRNA expression level of inflammatory genes and NF-κB transcriptional activity. (**A**) Viability of HEK293T cells treated with Mh-ME (0–200 μg/mL) investigated using the MTT assay. (**B**) Gene expression levels of iNOS, IL-6, and IL-1β measured by semi-quantitative RT-PCR in RAW264.7 cells, pretreated with Mh-ME (0, 50, 100, and 200 μg/mL), stimulated by LPS (1 μg/mL) for 6 h. (**C**,**D**) Transcriptional activity of NF-κB evaluated using the luciferase reporter gene assay in HEK293T cells transfected with FLAG-MyD88, CFP-TRIF, NF-κB-Luc, and β-galactosidase (as a transfection control) and treated with 100 and 200 μg/mL of Mh-ME. ## *p* < 0.01 compared with the group without transfection of adapter molecule gene; ** *p* < 0.01 relative to the control group.

We aimed to discover the specific molecular mechanism of the anti-inflammatory effect of Mh-ME. To determine the effect of Mh-ME on inflammatory gene expression at the transcriptional level, RAW264.7 cells pretreated with Mh-ME were stimulated by LPS (1 μg/mL). Then, we obtained cDNA from the cell lysates and checked the mRNA expression level of inflammatory agents using semi-quantitative RT-PCR. The results showed that Mh-ME (50, 100, and 200 μg/mL) downregulated the LPS-induced IL-6 and IL-1β transcription. Moreover, the iNOS gene levels were reduced by Mh-ME (100 and 200 μg/mL) treatment (Figure 3B). Thus, Mh-ME downregulated the expression of iNOS, IL-6, and IL-1β upregulated by LPS induction, confirming its effects on inflammatory activities.

Since Mh-ME regulates the expression of inflammatory genes, and NF-κB is a key transcription factor that proliferates the transcription of these genes, we examined whether Mh-ME could control this transcriptional factor using a luciferase assay. For this, the MTT assay was performed to evaluate the cell viability of HEK293T cells treated with Mh-ME. Treatment with 200 μg/mL of Mh-ME did not show cytotoxicity in HEK293T cells (Figure 3A). In the luciferase assay, Mh-ME (100 and 200 μg/mL) significantly reduced the luciferase activity mediated by NF-κB induced by MyD88 overexpression (Figure 3B). Moreover, Mh-ME (200 μg/mL) also significantly attenuated NF-κB-mediated luciferase activity induced by TRIF overexpression (Figure 3C,D). These results suggest that Mh-ME targets NF-κB signaling to exert anti-inflammatory properties.

### 2.4. Mh-ME Lowered Phosphorylation of Proteins Inducing the NF-κB Acitvation

**Figure 4:**
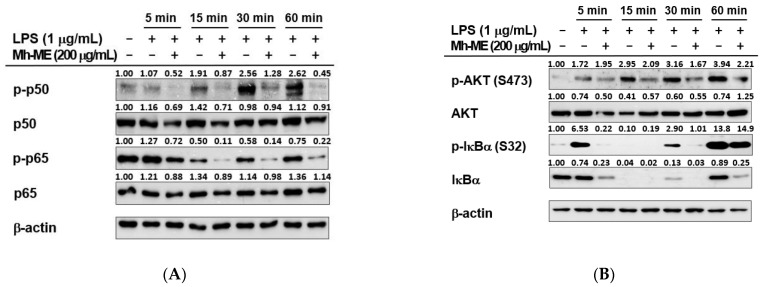

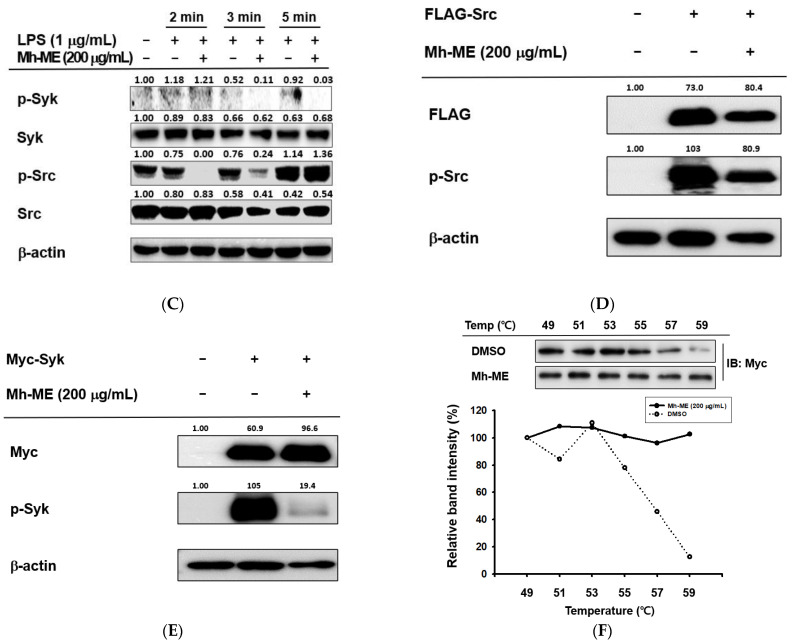
Effects of Mh-ME on the NF-κB signaling cascades. (**A**) Phosphorylation levels of NF-κB subunits, p50 and p65, evaluated by Western blotting in macrophages administered with Mh-ME (200 μg/mL) and stimulated with LPS (1 μg/mL). (**B**,**C**) Phosphorylation levels of NF-κB upstream molecules, AKT, IκBα, Src, and Syk, evaluated by Western blotting in RAW264.7 cells pretreated with Mh-ME (200 μg/mL) and stimulated with LPS (1 μg/mL). (**D**,**E**) Overexpression of Syk and Src in HEK293T cells using PEI transfection. HEK293T cells were transfected with FLAG-Src and Myc-Syk and treated with Mh-ME (200 μg/mL). (**F**) HEK293T cells treated with Mh-ME (100 µg/mL) or DMSO (as a control). HEK293T cells were transfected with Myc-Syk, and CETSA was conducted with anti-Myc antibodies. CETSA was conducted to analyze the binding capacity of Syk and Mh-ME. The relative band intensity values were obtained by dividing the values by the β-actin band intensity.

In previous results, we confirmed that Mh-ME inhibited the role of NF-κB as a transcription factor. Therefore, we investigated whether Mh-ME is related to other upstream signaling molecules associated with the NF-κB signaling pathway via conducting Western blotting analysis. First, Mh-ME (200 μg/mL) reduced phosphorylation of NF-κB p50 and NF-κB p65, which are NF-κB subunits in RAW264.7 cells, after LPS treatment (Figure 4A). In particular, LPS induction triggered an increase in the phosphorylation of p50 (5, 15, 30, 60 min) and p65 (5 min) subunits. However, treatment of Mh-ME significantly attenuated the phosphorylation of both p50 and p65 subunits at all time points. When confirming the phosphorylation level of the upstream signaling molecules of NF-κB by Western blotting, Mh-ME downregulated the phosphorylation of IκBα (5, 30, and 60 min) and AKT (15, 30, and 60 min) in RAW264.7 cells under LPS stimulation (Figure 4B). The IκBα immunoblot band was not observed after 15 min of treatment of LPS, which is consistent with previous reports showing that IκBα degradation occurs between 15 and 30 min after LPS processing in macrophages [18]. Short-term exposure of LPS induced the phosphorylation of Syk and Src kinases (Figure 4C). However, the enhanced phosphorylation of Src was decreased by Mh-ME (200 μg/mL) between 2 and 5 min of LPS exposure, and phosphorylation of Syk was inhibited between 3 and 5 min of LPS exposure. Furthermore, Mh-ME (200 μg/mL) reduced exogenous Syk and Src phosphorylation in HEK293T cells (Figure 4D,E). In particular, phosphorylation of exogenous Syk was dramatically decreased after Mh-ME treatment. Therefore, the CETSA assay was employed to determine whether the molecular target of Mh-ME is Syk kinase. The results showed that exogenous Syk was stabilized by Mh-ME between 51 and 61 °C, indicating that Mh-ME can increase the thermal stability of Syk by direct binding. These results suggest that the active component of Mh-ME directly binds with Syk and ameliorates NF-κB signaling.

### 2.5. Mh-ME Showed Therapeutic Potential on Acute Gastritis

**Figure 5:**
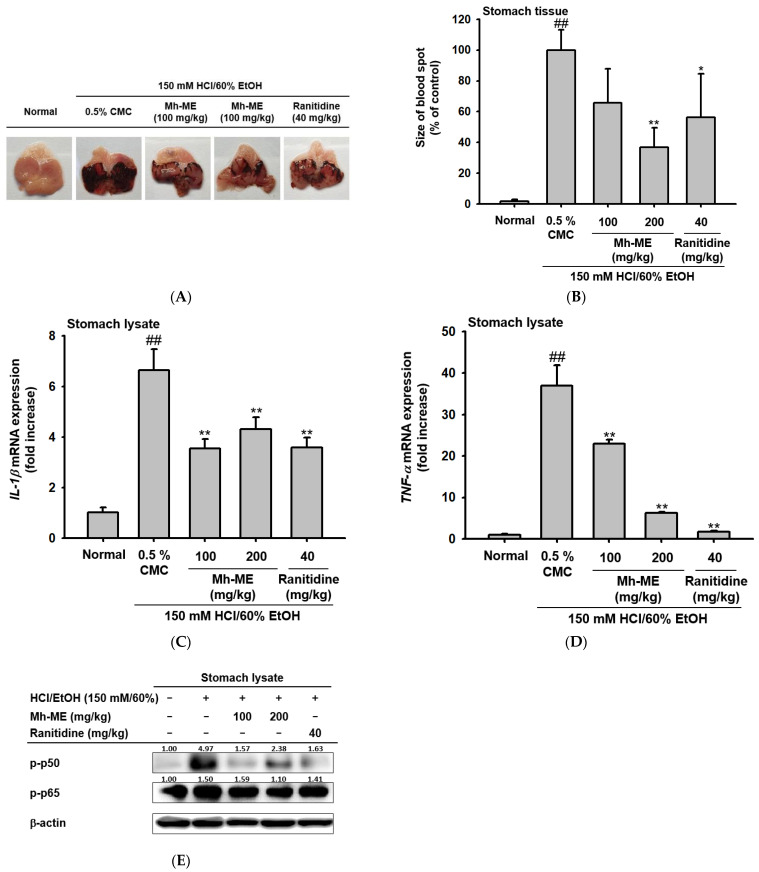
Effects of Mh-ME in HCl/EtOH-triggered gastritis-induced mice. (**A**,**B**) Mh-ME (100 and 200 mg/kg) was orally administered to mice, and acute gastritis was induced by 150 mM HCl/60% EtOH for 1 h. After sacrifice, pictures of inflammatory lesions in the stomach were collected, and the sizes of blood spots were quantitatively evaluated using ImageJ (ver. 1.8.0). (**C**,**D**) The mRNA expression levels of IL-1β and TNF-α from stomach lysate were examined by quantitative real-time PCR. (**E**) Phosphorylation levels of p50 and p65 from stomach lysates of mice were detected by Western blotting. All data from three independent experiments are represented as the mean ± SD value. ## *p* < 0.01 compared with the normal group; * *p* < 0.05 and ** *p* < 0.01 compared with the control group.

Additionally, the inflammation-alleviating effect of Mh-ME in an in vivo gastritis mouse model was evaluated. Gastritis in ICR mice was induced with 150 mM HCl/60% EtOH, and Mh-ME and ranitidine, a positive control drug, were administered three times at 12 h intervals. Thereafter, hemorrhagic mucosal damage was observed to determine the degree of inflammation. Both the ranitidine and Mh-ME administration groups showed decreased bleeding and redness of the gastric mucosa (Figure 5A,B). As with the results of the in vitro experiments, Mh-ME reduced the expression of pro-inflammatory cytokine in stomach tissue, upregulated by administration of 150 mM HCl/60% EtOH (Figure 5C,D). In specific, 200 mg/kg of Mh-ME drastically ameliorated the mRNA expression of IL-1β and TNF-α to the point of comparison to ranitidine. In addition, Mh-ME administration reduced the phosphorylated forms of p50 and p65 in stomach lysates from mice (Figure 5E).

### 2.6. Acute Toxicity Test

**Figure 6:**
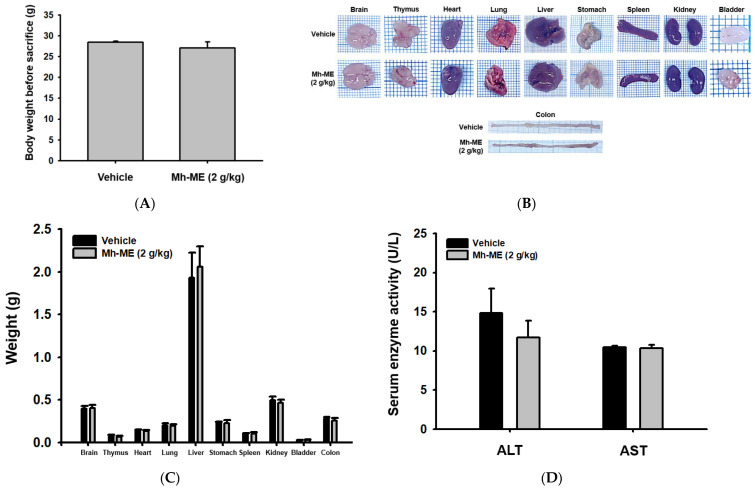
Toxicity test after oral administration of single dose of 2 g/kg of Mh-ME in ICR mice. (**A**) Body weight of ICR mice after administration of 2 g/kg of Mh-ME was measured. (**B**) Organ images were obtained from the mice. (**C**) The weight of each organ was analyzed. (**D**) Serum ALT and AST levels were determined from serum.

For assessment of toxicological effects of Mh-ME in mice, 10 time-dose units of Mh-ME (2 g/kg) were orally administered to ICR mice. Then, body and organ weights in the Mh-ME-treated group were compared with those of control (non-treated normal) mice with oral administration of 0.5% CMC. The body weight of ICR mice was not changed by oral administration of 2 g/kg of Mh-ME (Figure 6A). The size and weight of each organ also had little or no change after administration of Mh-ME, indicating that a high dose of Mh-ME has no acute toxicity to ICR mice (Figure 6B,C). The activity of alanine aminotransferase (ALT) and aspartate aminotransferase (AST) in serum, representing free radicals in hepatocytes, became a criterion of liver toxicity assessment [19,20]. Since reaction of ALT and AST, respectively, produces pyruvate and glutamate, we can measure the serum ALT and AST activity via measurement of pyruvate and glutamate. ALT and AST activity were maintained after 2 g/kg of Mh-ME treatment, suggesting that Mh-ME did not induce liver injury. Although long-term toxicity tests should be further conducted, these findings demonstrate that a single, high dose of Mh-ME did not result in toxic effects in mice.

## 3. Discussion

There are many anti-inflammatory agents used as commercial drugs. Most well-known drugs may include non-steroidal anti-inflammatory drugs (NSAIDs) such as ibuprofen [21]. However, NSAIDs mainly target cyclooxygenases (COXs) blocking the synthesis of prostaglandin, which is responsible for fever and pain [22,23]. COXs, especially COX-2, can be expressed by the transcriptional activity of NF-κB. This indicates that NSAIDs have a narrow point of application since they only suppress a single result of NF-κB signaling. Moreover, steroidal anti-inflammatory drugs like dexamethasone exert harmful side effects like muscle atrophy and stomach irritation [24]. Ranitidine, which was used as a positive control drug in our experiment, was removed from the pharmaceutical market since it induces a carcinogen called N-nitrosodimethylamine (NDMA) [25,26]. In this situation, there is a demand for the development of anti-inflammatory drugs with few side effects and a wide application point.

*Meriania hexamera* Sprague is a plant belonging to the Melastomataceae family, and plants from Melastomataceae family are known for rich phenolic compounds such as flavonoids, terpenoids, quinones, lignans, and tannins. For instance, fruits of *Miconia albicans* are rich in flavonoids and terpenoids, which exert antioxidant and anti-inflammatory properties [27]. *Meriania hernandoi* and *Meriania nobilis* leaf extracts contain tannins like merianin A and B, which have various therapeutic effects [13]. We hypothesized that *M. hexamera* may also have anti-inflammatory effects. However, no research has focused on the anti-inflammatory activity of *M. hexamera* and its specific molecular mechanism. Therefore, we investigated the anti-inflammatory activity of Mh-ME in vitro and in vivo using murine macrophage cells and human embryonic cells as well as a mouse model of gastritis. Also, we identified the Mh-ME target molecules in the inflammatory signaling pathway.

Inflammation has a major role in immune response, but it can worsen to chronic inflammation, which leads to multiple diseases, such as cancer and atherosclerosis. Nitric oxide (NO) is a representative indicator substance that can announce inflammatory responses [28]. NO induces dilation of blood vessels to help immune cells relocate to lesion areas so that immune cells can remove pathogens or damaged cells [29]. Because NO can be used as a marker of inflammatory response, in this study, we examined the NO production level of macrophages treated with Mh-ME. Mh-ME (0–200 μg/mL) diminished NO level in murine macrophage cell line stimulated by either LPS (1 μg/mL), Pam3CKS4 (10 μg/mL), or poly(I:C) (1 μg/mL) in a dose-dependent manner (Figure 1A–C) without altering the cell viability (Figure 1E). Interestingly, it has been found that Mh-ME consists of a high amount of pyrogallol, which showed a remarkable NO-reducing effect (Figure 2B). Since pyrogallol moiety is one of the active components of flavonoids, we assumed that Mh-ME also has an abundant number of flavonoids which may exert various therapeutic efficacies [30]. The follow-up study showed that Mh-ME contains high numbers of polyphenols, of which there are 121 different types of flavonoids.

Stimuli such as LPS, Pam3CSK4, and poly (I:C) damage cells or tissues and induce NO production accompanied with the activation of pro-inflammatory molecules like iNOS, IL-6, IL -1β, and TNF-α [31]. Consistent with previous results, these inflammatory genes are reduced by Mh-ME (50, 100, and 200 μg/mL) in LPS-stimulated RAW 264.7 cells, where they were especially hindered at 200 μg/mL, as evaluated using semi-quantitative reverse transcription PCR (Figure 3B). Therefore, we confirmed that Mh-ME exerts anti-inflammatory effects by blocking the expression of pro-inflammatory agents.

NF-κB is a major regulator of inflammatory genes, including iNOS, IL-6, and IL-1β genes. Thus, in this study, we investigated the effects of Mh-ME on the transcriptional activity of NF-κB by employing a luciferase reporter gene assay. Since it is difficult to move exogenous genes into the nucleus of RAW264.7 cells, we used HEK293T cells in the luciferase assay [32]. By overexpressing MyD88 and TRIF, which are intracellular adaptor proteins that transport signals from TLRs and activate the NF-κB signaling pathway, we created a condition similar to LPS stimulation [33]. Mh-ME significantly suppressed the activity of NF-κB-Luc induced by both MyD88 (100 and 200 μg/mL of Mh-ME) and TRIF (200 μg/mL of Mh-ME) in the luciferase reporter gene assay (Figure 3B,C). Moreover, NF-κB consists of p50/p65 heterodimers, where phosphorylation of these subunits affects the NF-κB transcriptional activity, and Mh-ME (200 μg/mL) controls the phosphorylation of p50 and p65 (Figure 4A) [34]. Effective suppression of Mh-ME in p50 and p65 indicates that Mh-ME decreases inflammatory genes by reducing the activity of NF-κB.

The NF-κB pathway is highly associated with the inflammation response and disease [35]. Thus, inhibition of the NF-κB signaling pathway has a significant effect on reducing the inflammatory response. Determining the exact target of Mh-ME is crucial for therapeutic strategies developed using Mh-ME. Thus, we observed AKT, IκBα, and upstream protein kinase Src/Syk, which are representative signal transmitters for the NF-κB pathway. When cells are not stimulated, NF-κB exists as an inactive dimer in the cytoplasm. However, within the stimulus, NF-κB is activated by proteasomal degradation of IκB, an NF-κB protein inhibitor [36]. IκB phosphorylation is induced by IκB kinase (IKK), which is activated by AKT [37]. In particular, Src and Syk kinases are upstream enzymes that regulate downstream molecules such as PI3K, PDK1, and AKT in the TLR4 signaling cascade [38,39]. Inhibition of these enzymes has been reported to suppress the inflammatory symptoms induced by NF-κB. Mh-ME effectively suppressed phosphorylation of IκBα, Akt, Src, and Syk (Figure 4B,C). Furthermore, Mh-ME impressively inhibited the activation of Syk in the overexpression condition (Figure 4D,E). Moreover, in the CETSA analysis, Mh-ME strongly binds to Syk as its molecular target (Figure 4F). Based on our findings, Mh-ME has an anti-inflammatory effect by targeting Src and Syk in the NF-κB signal pathway. Since Mh-ME successfully reduced the inflammatory lesion in the gastritis mouse model (Figure 5A,B) and a single dose of Mh-ME had no toxic effect on ICR mice (Figure 6), we can emphasize the pharmaceutical role of Mh-ME as an anti-inflammatory medicine.

Taken together, these results suggest that Mh-ME directly targets Syk kinase and subsequently downregulates NF-κB signaling cascades to exert anti-inflammatory effects with no cytotoxicity. Mh-ME also showed promising effects on ameliorating acute gastritis. As it is a plant extract, standardization of the active compounds should be conducted, and it is necessary to find out whether Mh-ME has remarkable efficacy in clinical trials. For this, we will first continue to perform toxicological evaluation as well as explore other pharmacological efficacies of this extract to improve druggable or functional food value.

## 4. Materials and Methods

### 4.1. Materials and Reagents

Roswell Park Memorial Institute 1640 (RPMI 1640), Dulbecco’s modified Eagle’s medium (DMEM), fetal bovine serum, streptomycin, and penicillin were purchased from GIBCO (Grand Island, NY, USA). Lipopolysaccharide (LPS), Poly (I:C:), Pam3CSK4, 3-(4-5-dimethylathiaol-2-yl)-2-5-diphenyltetrazolium bromide (MTT), L-NAME (NO inhibitor), dimethyl sulfoxide (DMSO), sodium dodecyl sulfate (SDS), and ranitidine were purchased from Sigma-Aldrich (St. Louis, MO, USA). Pyrogallol was purchased from MedChemExpress (Monmouth Junction, NJ, USA). Phosphate-buffered saline (PBS) was received from Samchun Pure Chemical Co. (Gyeonggi-do, Republic of Korea). TRIzol was purchased from Molecular Research Center, Inc. (Cincinnati, OH, USA). The cDNA synthesis kit was purchased from Thermo Fisher Scientific (Waltham, MA, USA), and primers for semiquantitative RT-PCR and quantitative real-time PCR were procured from Macrogen (Seoul, Republic of Korea). The PCR premix was received from Bio-D Inc. (Seoul, Republic of Korea). Antibodies against β-actin were obtained from Santa Cruz Biotechnology, Inc. (Santa Cruz, CA, USA). Total-protein and phospho-specific p50, p65, IκBα, AKT, Syk, Src, and Myc targeting antibodies were produced by Cell Signaling Technology (Beverly, MA, USA).

### 4.2. Mh-ME Preparation and GC-MS

Mh-ME, prepared from the leaves and branches of M. hexamera, was purchased from the International Biological Material Research Center (Daejeon, Republic of Korea) in a completely freeze-dried form after the solvent (methanol) was removed. The Mh-ME powder was diluted in DMSO to obtain 200 mg/mL of stock solution. For animal experiments, Mh-ME was dissolved in a 0.5% sodium carboxymethylcellulose (CMC) solution at a concentration of 200 mg/mL. GC-MS was performed with the cooperation of Cooperative Center for Research Facilities at SKKU (Suwon, Republic of Korea). Specifically, gas chromatography was conducted using Agilent 8890GC equipment with a J&W DB-624 Ultra Insert gas chromatography column (Santa Clara, CA, USA). A total of 1 μL of 100 mg/mL Mh-ME was inserted into the column, and the operation time was 40 min. Mass spectrometry was conducted with Agilent 5977B MSD equipment (Santa Clara, CA, USA) for 10 min. Based on the phytochemical spectra from the National Institute of Standards and Technology library, phytochemicals in Mh-ME were identified.

### 4.3. Measurement of Total Phenolic and Flavonoid Contents

To detect the phenolic compounds in Mh-ME, the method from Elizabeth A Ainsworth and Kelly M Gillespie was adopted [40]. A total of 100 μL of Mh-ME (15× concentration) and 100 μL of 10% Folin–Ciocalteu reagent were mixed in 300 μL of distilled water for 5 min in RT. Then, 1 mL of 4% sodium carbonate diluted in distilled water was added to the mixture. As the gallic acid derivatives turned the color of the Folin–Ciocalteu reagent from yellow to dark blue, absorbance was measured at 765 nm. Gallic acid was used to draw a calibration curve.

The total flavonoid content of Mh-ME was detected with the method previously described [41]. A total of 50 μL of Mh-ME (2x concentration) diluted in methanol was added to 50 μL of 2% aluminum chloride dissolved in methanol. Subsequently, the solution was incubated for 1 h in RT. The absorbance of the solution was measured at 420 nm. Quercetin was used to obtain a standard curve.

### 4.4. LC-MS/MS

Flavonoid profiles of Mh-ME were analyzed by a Xevo G2-Xs LC-qTOF-MS/MS (Walters, Milford, MA, USA). A total of 0.1% formic acid in distilled water (A) and acetonitrile (B) were used as the mobile phase. The UPLC column (BEH C18, 2.1 × 100 mm, 1.7 mm) was used as the reverse phase. In total, 2 μL of 100 mg/mL of Mh-ME was injected to the column with the flow rate of 300 μL/min in 45 °C. The mass spectrometry was performed to detect the 40–12,000 *m*/*z* range with +ESI mode. The data were collected and reorganized with Waters LC-qTOF-MS/MS MassLynx software version 4.2 and Waters UNIFI portal software, respectively.

### 4.5. Cell Culture

The RAW264.7 cell line (mouse-derived macrophage) was bought from the Korean Cell Line Bank (Seoul, Republic of Korea), and the HEK293T cell line (Human embryonic kidney cell) was purchased from American Type Culture Collection (ATCC) (Manassas, VA, USA). The RAW264.7 cell line was cultured in an RPMI 1640 medium, and the HEK293T cell line was cultured in DMEM medium at 37 °C under 5% CO_2_ in an incubator. Each of the media was established with 10% FBS (RPMI 1640) or 5% FBS (DMEM). A 1% penicillin-streptomycin solution was added to both media to avoid contamination. The HEK293T cells were detached by Trypsin for subculturing, while the RAW2647 cells were obtained using a cell scraper.

### 4.6. Nitric Oxide (NO) Assay

The RAW264.7 cell line at 1 × 10^6^ cells/mL was pretreated with Mh-ME or L-NAME for 30 min with the subsequent exposure of LPS for 24 h. The resulting supernatant (100 μL) was mixed with 100 μL of Griess reagent, as previously reported [42]. The absorbance was then detected at 540 nm. NO concentration was calculated by the converting the absorbance value with the standard curve.

### 4.7. Cell Viability Assay

RAW264.7 cells and HEK293T cells with a cell density of 1 × 10^6^ cells/mL were dose-dependently treated with Mh-ME for 24 h. Then, as previously reported, 10 μL of the MTT solution was added, incubated for 3 h, and stopped in 15% SDS [43]. The absorbance of MTT formazan was measured at 570 nm.

### 4.8. Semiquantitative RT-PCR and Quantitative Real-Time PCR

RAW264.7 cells with a cell density of 1 × 10^6^ cells/mL pretreated with Mh-ME for 30 min were treated with LPS (1 μL) for 6 h. The cells were lysed with TRIzol reagent, and mRNA was extracted for cDNA synthesis. In semiquantitative RT-PCR, the mRNA expression levels of iNOS, IL-1β, IL-6, and GADPH from cDNA were obtained in RAW264.7 cells. In the in vivo acute gastritis model, the stomach of the mouse was ground with liquid N_2_. The total RNA from tissues was also isolated with TRIzol reagent and used for cDNA synthesis. In quantitative real-time PCR, the mRNA expression levels of IL-1β and TNF-α were quantified based on the expression of glyceraldehyde 3-phosphate dehydrogenase (GAPDH). Table 4 and Table 5 provide the lists of primers.

### 4.9. Luciferase Reproter Assay

HEK293T cells with a cell density of 1 × 10^6^ cells/mL were loaded onto 24-well plates and cultured for 24 h. Afterward, plasmids containing the luciferase gene with NF-κB binding response elements were introduced to HEK293T cells. MyD88 and TRIF genes, to activate the luciferase gene, and β-galactosidase genes to measure the transfection efficacy, were also co-transfected to HEK293T cells. The transfection process was assisted by polyethylenimine (PEI) [43]. At 24 h after transfection, Mh-ME was introduced to the cells for another 24 h. After cell lysis with a freeze-thaw cycle, the activity of luciferase from cell lysates was measured by treating luciferin as a substrate.

### 4.10. Preparing Whole Lysates and Western Blotting

RAW264.7 cells and HEK293T cells were washed with cold PBS. Then, cell pellets were obtained from centrifugation, and the pellets were lysed with subsequent sonication in cell lysis buffer (50 mM Tris-HCl, pH 7.4, 1% NP-40, 0.25% sodium deoxycholate, 150 mM NaCl, 1mM Na_3_VO_4_, and 1 mM NaF) supplemented with protease inhibitor cocktail (100 μg/mL leupeptin, 10 μg/mL pepstatin, 1 μg/mL aprotinin, 2 mM EDTA, and 2 mM PMSF) for 30 min. The obtained lysates were centrifuged at 13,000 rpm for 10 min at 4 °C. Cell lysates were inspected using 10% SDS-polyacrylamide gel electrophoresis. Proteins from the polyacrylamide gel were moved onto a polyvinylidene difluoride (PVDF) membrane that then was blocked with 3% BSA in tris-buffered saline with Tween 20 (TBST) to protect the specific antibody interactions. The membrane was incubated with primary antibody in 3% BSA in TBST for more than 3 h. Then, the membrane was washed with TBST three times and reacted with a secondary antibody in 3% BSA in TBST for 1 h. The immunoreactivity was detected with an enhanced chemiluminescence kit (Pierce ECL Western blotting substrate, Thermo Scientific, Waltham, MA, USA). The band intensity was quantified with image J software.

### 4.11. Plasmid Transfection for Exogeneous Expression of Syk and Src Kinase

HEK293T cells were plated at 1 × 10^6^ cells/mL in 6-well plates and transfected with HA-Src and Myc-Syk genes for 24 h using PEI. Subsequently, the cells were treated with Mh-ME and incubated for 24 h. The cells were lysed, and protein samples were obtained for the Western blotting analysis.

### 4.12. Cellualr Thermal Shift Assay (CETSA)

HEK293T cells were plated in a 6 cm plate and cultured for 24 h. Then, the cells were transfected with Myc-Syk using PEI, as previously reported [43], and treated with Mh-ME for 24 h. The CETSA assay was then conducted as reported previously [44]. Western blotting analysis was conducted to measure the band intensity of Myc protein to indicate the degree of thermal decomposition of exogenous Syk kinase.

### 4.13. Animal Experiments

ICR mice (male, 5 weeks old) were provided by Orient Bio (Seoul, Republic of Korea). We only used male mice because females have the risk that each may have a greater diversity due to estrous cycles, as previous studies with the gastritis model discussed [45]. The mice were acclimatized at a 12 h day–night cycle in a pathogen-free condition. All mice have free access to water and diet ad libitum (Samyang, Daejeon, Republic of Korea). We followed the instructions of the Institutional Animal Care and Use Committee at Sungkyunkwan University (Suwon, Republic of Korea, approval ID: SKKUIACUC2021-05-42-1) for the animal rights. Different doses of Mh-ME (0–200 mg/kg) or 40 mg/kg of ranitidine dissolved in 0.5% CMC were orally administered three times for two days with a 12 h interval. Acute gastritis occurred in the mice with an oral administration of 300 μL of 60% EtOH in 150 mM HCl, as previously described [46]. During the sacrifice process, the mice were anesthetized by CO_2_ gas. The stomach was extracted and washed with PBS. Then, the areas connected to the esophagus and duodenum were incised to create a widened image of the stomach for analysis of inflammatory lesions. After obtaining the images, the tissues were lysed with liquid nitrogen and stored at −70 °C for further analysis.

For the acute toxicity test, ten 5-week-old male mice were purchased and randomly assigned to two groups: vehicle group and Mh-ME treatment group. One mL of 0.5% CMC was orally administered to mice from the vehicle group, while 1 mL of 2 g/kg of Mh-ME diluted in 0.5% CMC was administered to those from the Mh-ME group. Twenty-four hours after oral injection, body weight of the mice was analyzed, and all mice were sacrifice with CO_2_ gas. Organs including bladder, blood, brain, colon, heart, kidney, liver, lung, spleen, stomach, and thymus were obtained from the mice.

### 4.14. Serum ALT and AST Activity Detection

Blood of mice from the acute toxicity test were centrifuged at 3000 rpm for 15 min to obtain serum. Colorimetric ALT and AST assay kits (abcam, Cambridge, UK) were used to detect the level of serum pyruvate and glutamate with the manufacture’s protocols. The pyruvate level was measured at 570 nm, and the glutamate level was measured at 450 nm using spectrophotometer.

### 4.15. Statistical Anaylsis

All experiments were triplicated to confirm the reproducibility of the measurements, and the data are shown as mean ± SD value. All data from the in vitro experiments were evaluated by one-way ANOVA with the Tukey’s multiple comparisons test. Statistical significances from the in vivo experiments were determined by one-way ANOVA, with Dunnett’s multiple comparisons test evaluating significant differences with the LPS induction group. GraphPad Prism 8.01 software from GraphPad Software (La Jolla, CA, USA) was utilized for the calculations. A *p*-value < 0.05 showed statistical significance.

## 5. Conclusions

**Figure 7:**
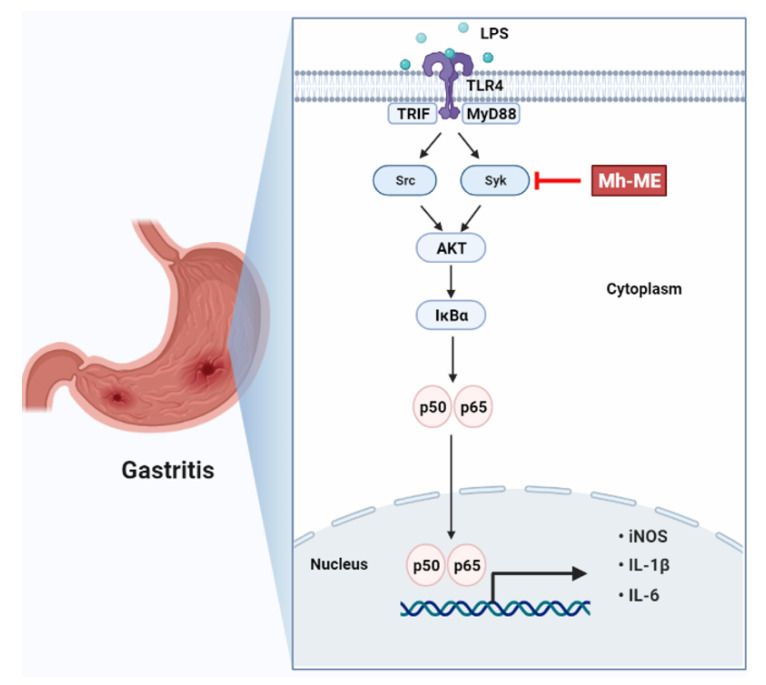
Schematic of the anti-inflammatory mechanisms of Mh-ME targeting Syk in the NF-ĸB signaling pathway.

Mh-ME suppressed the expression of inflammation in RAW264.7 cells and suppressed the symptoms of gastritis in HCl/EtOH-induced gastritis. In addition, Mh-ME inhibited the activation of p50, p65, IĸBα, AKT, and Syk, which are LPS-induced signal transmitters. Taken together, these findings demonstrate an anti-inflammatory effect in Mh-ME, as summarized in Figure 7. Gastritis is a disease that damages the gastric mucosa, causing abdominal pain and heartburn, and can develop into gastric cancer. Current drug development focuses on multitarget drugs. In particular, the importance of drug development of natural products that can interact with various proteins is growing. In this regard, the anti-inflammatory effect of Mh-ME extracted from traditional plants can be expected to be of great help in the development of anti-inflammatory drugs.

## Figures and Tables

**Table 1:** GC-MS analysis of phytochemical from Mh-ME.

Peak No.	RT (min)	Compound Name	Corrected Area	% of Total
1	8.780	5-Hydroxymethylfurfural	5,310,668	1.388
2	9.806	1,1-diethyl-2-(1-methyl propyl)-Hydrazine	7,381,173	1.929
3	10.301	2,7-Oxepanedione	4,908,751	1.283
4	10.810	1,2,3-Benzenetriol	251,749,615	65.794
5	11.193	1,2,3-Benzenetriol	5,064,771	1.324
6	12.231	1,6-anhydro-beta-D-Glucopyranose	28,492,675	7.446
7	13.946	4-hydroxy-Benzenepropanoic acid	7,835,372	2.048
8	14.221	4-hydroxy-3-methoxy- Benzenepropanol	7,587,564	1.983
9	16.218	Neophytadiene	4,036,831	1.055
10	16.663	Z-8-Methyl-9-tetradecen-1-ol formate	2,380,805	0.622
11	17.410	n-Hexadecanoic acid	20,611,816	5.387
12	19.114	(Z,Z,Z)-9,12,15-Octadecatrienoic acid	9,550,118	2.496
13	19.299	Octadecanoic acid	2,647,456	0.692
14	29.383	gamma-Sitosterol	2,5075,899	6.554

**Table 2:** Total flavonoid and phenolic contents of Mh-ME.

Extract	Total Phenolic Contents ^1^	Total Flavonoid Contents ^2^
Mh-ME	301.33 ± 0.98	19.05 ± 1.19

^1^ Representing the amount of gallic acid derivatives in Mh-ME (mg/g). ^2^ Representing the amount of quercetin in Mh-ME (mg/g).

**Table 3:** Phenolic and flavonoid compounds with anti-inflammatory properties analyzed by LC-MS from Mh-ME.

RT (min)	Component Name	Formula
0.94	Wogonoside	C_22_H_20_O_11_
0.98	Kaempferol-7-O-α-L-rhamnoside	C_21_H_20_O_10_
2.65	Bavachinin	C_21_H_22_O_4_
2.90	Kaempferol-3-gentiobioside	C_27_H_30_O_16_

**Table 4:** Sequences of primers used for semi-quantitative RT-PCR analysis.

Gene Name		Sequence (5′-3′)
iNOS	Forward Reverse	TGCCAGGGTCACAACTTTACA ACCCCAAGCAAGACTTGGAC
IL-1β	Forward Reverse	CAGGATGAGGACATGAGCACC CTCTGCAGACTCAAACTCCAC
IL-6	Forward Reverse	GGAAATCGTGGAAATGAG GCTTAGGCATAACGCACT
GAPDH	Forward Reverse	GAAGGTCGGTGTGAACGGAT AGTGATGGCATGGACTGTGG

**Table 5:** Sequences of primers used for quantitative real-time PCR analysis.

Gene Name		Sequence (5′-3′)
IL-1β	Forward Reverse	GTGAAATGCCACCTTTTACAGTG CCTGCCTGAAGCTCTTGTTG
TNF-α	Forward Reverse	TGCCTATGTCTCAGCCTCTT GAGGCCATTTGGGAACTTCT
GAPDH	Forward Reverse	GGAGAGTGTTTCCTCGTCCC ATGAAGGGGTCGTTGATGGC

## Data Availability

The data used to support the findings of this study are available from the corresponding author upon request.

## Supplementary Materials

The following supporting information can be downloaded at: https://www.mdpi.com/article/10.3390/plants12173044/s1, Table S1: LC-MS/MS analysis of phenolic and flavonoid compounds from Mh-ME.

## Abbreviation

PAMPs	pathogen-associated molecular patterns
PRRs	pattern recognition receptors
TLRs	toll-like receptors
LPS	lipopolysaccharide
TRIF	TIR-domain-containing adapter-inducing interferon-β
MyD88	myeloid differentiation response gene 88
DAMPs	damage-associated molecular patterns
NF-κB	nuclear factor-kappa B
AP-1	activated protein-1
Syk	spleen tyrosinase kinase
AKT	protein kinase B
IκBα	inhibitor of kappa B alpha
iNOS	nitric oxide synthase
IL-1β	interleukin-1β
TNF-α	tumor necrosis factor-alpha
COX-2	cyclooxygenase-2
Mh-ME	*M. hexamera* Sprague-Methanol Extract
Aa-EE	*A. asiatica*-Ethanol Extract
GC-MS	gas chromatography-mass spectrometry
RPMI 1640	Roswell Park Memorial Institute 1640
DMEM	Dulbecco’s modified Eagle’s medium
MTT	3-(4-5-dimetyhlathiaol-2-yl)-2-5-diphenyltetrazolium bromide
DMSO	dimethyl sulfoxide
SDS	sodium dodecyl sulfate
PBS	phosphate-buffered saline
CMC	carboxymethylcellulose
GAPDH	glyceraldehyde 3-phosphate dehydrogenase
PEI	polyethylenimine
TBST	tris-buffered saline with Tween 20
CETSA	cellular thermal shift assay
ALT	alanine aminotransferase
AST	aspartate aminotransferase

## References

[1] Fujiwara N., Kobayashi K. (2005). Macrophages in inflammation. Curr. Drug Targets Inflamm. Allergy.

[2] Coussens L.M., Werb Z. (2002). Inflammation and cancer. Nature.

[3] Ratan Z.A., Rabbi Mashrur F., Runa N.J., Kwon K.W., Hosseinzadeh H., Cho J.Y. (2022). Ginseng, a promising choice for SARS-COV-2: A mini review. J. Ginseng Res..

[4] Medzhitov R., Preston-Hurlburt P., Janeway C.A. (1997). A human homologue of the Drosophila Toll protein signals activation of adaptive immunity. Nature.

[5] Da Silva Correia J., Soldau K., Christen U., Tobias P.S., Ulevitch R.J. (2001). Lipopolysaccharide is in close proximity to each of the proteins in its membrane receptor complex. J. Biol. Chem..

[6] Ullah M.O., Sweet M.J., Mansell A., Kellie S., Kobe B. (2016). TRIF-dependent TLR signaling, its functions in host defense and inflammation, and its potential as a therapeutic target. J. Leuk. Biol..

[7] Tak P.P., Firestein G.S. (2001). NF-κB: A key role in inflammatory diseases. J. Clin. Investig..

[8] Lee Y.G., Chain B.M., Cho J.Y. (2009). Distinct role of spleen tyrosine kinase in the early phosphorylation of inhibitor of κBα via activation of the phosphoinositide-3-kinase and Akt pathways. Int. J. Biochem. Cell Biol..

[9] Lu Y.-C., Yeh W.-C., Ohashi P.S. (2008). LPS/TLR4 signal transduction pathway. Cytokine.

[10] Eming S.A., Krieg T., Davidson J.M. (2007). Inflammation in wound repair: Molecular and cellular mechanisms. J. Investig. Dermatol..

[11] Libby P., Ridker P.M., Hansson G.K. (2009). Inflammation in atherosclerosis. J. Am. Coll. Cardiol..

[12] Cruz A.d.M., Kaplan M. (2014). Medicinal uses of species from Myrtaceae and Melastomataceae families in Brazil. Floresta Ambiente.

[13] Valverde Malaver C.L., Colmenares Dulcey A.J., Rial C., Varela R.M., Molinillo J.M.G., Macías F.A., Isaza Martínez J.H. (2019). Hydrolysable tannins and biological activities of *Meriania hernandoi* and *Meriania nobilis* (Melastomataceae). Molecules.

[14] Jeong D., Yi Y.S., Sung G.H., Yang W.S., Park J.G., Yoon K., Yoon D.H., Song C., Lee Y., Rhee M.H. (2014). Anti-inflammatory activities and mechanisms of *Artemisia asiatica* ethanol extract. J. Ethnopharmacol..

[15] Yang Y.Z., Tang Y.Z., Liu Y.H. (2013). Wogonoside displays anti-inflammatory effects through modulating inflammatory mediator expression using RAW264.7 cells. J. Ethnopharmacol..

[16] You L., Cho J.Y. (2021). The regulatory role of Korean ginseng in skin cells. J. Ginseng Res..

[17] Deng H., Jiang J., Shu J., Huang M., Zhang Q.L., Wu L.J., Sun W.K. (2023). Bavachinin ameliorates rheumatoid arthritis inflammation via PPARG/PI3K/AKT signaling pathway. Inflammation.

[18] Ichikawa D., Matsui A., Imai M., Sonoda Y., Kasahara T. (2004). Effect of various catechins on the IL-12p40 production by murine peritoneal macrophages and a macrophage cell line, J774.1. Biol. Pharm. Bull..

[19] Kresnamurti A., Rakhma D.N., Damayanti A., Santoso S.D., Restryarto E., Hadinata W., Hamid I.S. (2021). AST/ALT levels, MDA, and liver histopathology of *Echinometra mathaei* ethanol extract on paracetamol-induced hepatotoxicity in rats. J. Basic Clin. Physiol. Pharmacol..

[20] Tahmasebi M., Sadeghi H., Nazem H., Kokhdan E.P., Omidifar N. (2018). Hepatoprotective effects of *Berberis vulgaris* leaf extract on carbon tetrachloride-induced hepatotoxicity in rats. J. Educ. Health Promot..

[21] Barbagallo M., Sacerdote P. (2019). Ibuprofen in the treatment of children’s inflammatory pain: A clinical and pharmacological overview. Minerva. Pediatr..

[22] Vane J.R., Botting R.M. (1998). Anti-inflammatory drugs and their mechanism of action. Inflamm. Res..

[23] García-Rayado G., Navarro M., Lanas A. (2018). NSAID induced gastrointestinal damage and designing GI-sparing NSAIDs. Expert Rev. Clin. Pharmacol..

[24] Wang L., Jiao X.F., Wu C., Li X.Q., Sun H.X., Shen X.Y., Zhang K.Z., Zhao C., Liu L., Wang M. (2021). Trimetazidine attenuates dexamethasone-induced muscle atrophy via inhibiting NLRP3/GSDMD pathway-mediated pyroptosis. Cell Death Discov..

[25] White C.M., Hernandez A.V. (2021). Ranitidine and Risk of N-Nitrosodimethylamine (NDMA) Formation. JAMA.

[26] Kantor E.D., O’Connell K., Du M., Mendelsohn R.B., Liang P.S., Braunstein L.Z. (2021). Ranitidine use and cancer risk: Results from UK biobank. Gastroenterology.

[27] Corrêa J.G.S., Bianchin M., Lopes A.P., Silva E., Ames F.Q., Pomini A.M., Carpes S.T., de Carvalho Rinaldi J., Cabral Melo R., Kioshima E.S. (2021). Chemical profile, antioxidant and anti-inflammatory properties of *Miconia albicans* (Sw.) Triana (Melastomataceae) fruits extract. J. Ethnopharmacol..

[28] Tripathi P., Tripathi P., Kashyap L., Singh V. (2007). The role of nitric oxide in inflammatory reactions. FEMS Immunol. Med. Microbiol..

[29] Korhonen R., Lahti A., Kankaanranta H., Moilanen E. (2005). Nitric oxide production and signaling in inflammation. Curr. Drug Targets Inflamm. Allergy.

[30] Furuno K., Akasako T., Sugihara N. (2002). The contribution of the pyrogallol moiety to the superoxide radical scavenging activity of flavonoids. Biol. Pharm. Bull..

[31] Molloy R.G., Mannick J.A., Rodrick M.L. (2005). Cytokines, sepsis and immunomodulation. Br. J. Surg..

[32] Stacey K.J., Sweet M.J., Hume D.A. (1996). Macrophages ingest and are activated by bacterial DNA. J. Immunol..

[33] Laird M.H.W., Rhee S.H., Perkins D.J., Medvedev A.E., Piao W., Fenton M.J., Vogel S.N. (2009). TLR4/MyD88/PI3K interactions regulate TLR4 signaling. J. Leuk. Biol..

[34] Christian F., Smith E., Carmody R. (2016). The Regulation of NF-κB subunits by phosphorylation. Cells.

[35] Yamamoto Y., Gaynor R.B. (2001). Therapeutic potential of inhibition of the NF-κB pathway in the treatment of inflammation and cancer. J. Clin. Investig..

[36] Oeckinghaus A., Ghosh S. (2009). The NF-kB family of transcription factors and its regulation. Cold Spring Harb. Perspect. Biol..

[37] Lu P., Chen J., Yan L., Yang L., Zhang L., Dai J., Hao Z., Bai T., Xi Y., Li Y. (2019). RasGRF2 promotes migration and invasion of colorectal cancer cells by modulating expression of MMP9 through Src/Akt/NF-κB pathway. Cancer Biol. Ther..

[38] Lee H.S., Moon C., Lee H.W., Park E.-M., Cho M.-S., Kang J.L. (2007). Src Tyrosine kinases mediate activations of NF-κB and integrin signal during lipopolysaccharide-induced acute lung injury. J. Immunol..

[39] Lowell C.A. (2011). Src-family and Syk kinases in activating and inhibitory pathways in innate immune cells: Signaling cross talk. Cold Spring Harb. Perspect. Biol..

[40] Ainsworth E.A., Gillespie K.M. (2007). Estimation of total phenolic content and other oxidation substrates in plant tissues using Folin-Ciocalteu reagent. Nat. Protoc..

[41] Lee H.P., Kim D.S., Park S.H., Shin C.Y., Woo J.J., Kim J.W., An R.B., Lee C., Cho J.Y. (2022). Antioxidant capacity of *Potentilla paradoxa* Nutt. and its beneficial effects related to anti-Aging in HaCaT and B16F10 Cells. Plants.

[42] Choi W., Kim H.S., Park S.H., Kim D., Hong Y.D., Kim J.H., Cho J.Y. (2022). Syringaresinol derived from *Panax ginseng* berry attenuates oxidative stress-induced skin aging via autophagy. J. Ginseng Res..

[43] Kim J.K., Shin K.K., Kim H., Hong Y.H., Choi W., Kwak Y.S., Han C.K., Hyun S.H., Cho J.Y. (2021). Korean Red Ginseng exerts anti-inflammatory and autophagy-promoting activities in aged mice. J. Ginseng Res..

[44] Lee J.O., Hwang S.H., Shen T., Kim J.H., You L., Hu W., Cho J.Y. (2021). Enhancement of skin barrier and hydration-related molecules by protopanaxatriol in human keratinocytes. J. Ginseng Res..

[45] Painsipp E., Wultsch T., Shahbazian A., Edelsbrunner M., Kreissl M.C., Schirbel A., Bock E., Pabst M.A., Thoeringer C.K., Huber H.P. (2007). Experimental gastritis in mice enhances anxiety in a gender-related manner. Neuroscience.

[46] Mitra A., Rahmawati L., Lee H.P., Kim S.A., Han C.K., Hyun S.H., Cho J.Y. (2022). Korean Red Ginseng water extract inhibits cadmium-induced lung injury via suppressing MAPK/ERK1/2/AP-1 pathway. J. Ginseng Res..

