# Supplementary material for: Anti-Inflammatory Effect of Meriania hexamera Sprague by Targeting Syk Kinase in NF-κB Signaling
Source: Plants (Basel). 2023 Aug 24;12(17):3044. doi: 10.3390/plants12173044 (PMC10490091; doi:10.3390/plants12173044)
Supplement: Supplementary file 1 [file plants-12-03044-s001.zip › plants-2503836-supplementary.pdf]

Table S1. LC-MS/MS analysis of phenolic and flavonoid compounds from Mh-ME.

RT (min)	Component Name	Formula	Mass Error (ppm)	Total Fragments Found	Isotope Match Mz RMS PPM	Isotope Match Intensity RMS Percent	Detector Counts	Response	Adducts	Neutral Mass (Da)	Observed Neutral Mass (Da)
0.73	Luteolin-7-β-D-glucopyranoside	C21H20O11	-1.9	12	7.29	5.31	64079	43131	+Li	448.10056	448.0997
0.92	5-Methylkaempferol	C16H12O6	-1.4	37	15.98	5.64	31383	24641	+Li	300.06339	300.0630
0.93	Rhamnocitrin-3-O-β-D-glucoside	C22H22O11	1.1	136	23.43	6.08	129002	41881	+Li	462.11621	462.1167
0.94	Wogonoside	C22H20O11	0.6	126	157.28	20.04	88502	1136	+K	460.10056	460.1009
0.94	Ombuin 3-O-β-D-glucoside	C23H24O12	0.2	156	4.77	43.43	174702	53430	+Li	492.12678	492.1269
0.94	OnjixanthoneⅡ	C15H12O7	-2.3	26	11.99	11.30	57749	10236	+Li	304.05830	304.0576
0.94	1,5-Dihydroxy-2,3,4,7-tetramethoxyxanthone	C17H16O8	0.4	31	11.94	2.08	42613	15364	+Li	348.08452	348.0847
0.95	Noririsflorentin	C19H16O8	1.9	41	9.59	153.41	13668	1924	+Li	372.08452	372.0853
0.96	Wogonin 7-O-glucuronide methyl ester	C23H22O11	0.9	148	31.97	34.00	117945	26013	+Na	474.11621	474.1166

0.96	Viscum neoside Ⅱ	$C_{25}H_{26}O_{13}$	-3.0	192	4.38	48.65	125320	6418	+Li	534.13734	534.1357
0.98	Kaempferol-7-O-α-L-rhamnoside	$C_{21}H_{20}O_{10}$	0.7	123	12.01	6.50	54923	31771	+Li	432.10565	432.1059
1.02	2,3-Dihydroiriegnin	$C_{18}H_{18}O_{8}$	1.8	6	2.59	45.86	10460	8394	+Li	362.10017	362.1008
1.03	Wogonoside (Wogonin-7-O-β-D-glucuronide)	$C_{22}H_{22}O_{10}$	-0.6	16	1.79	184.04	106054	40912	+Li	446.12130	446.1210
1.04	Homoplantaginin	$C_{22}H_{22}O_{11}$	0.7	27	1.34	346.63	124865	75987	+Li, +Na	462.11621	462.1165
1.05	Artemitin	$C_{20}H_{20}O_{8}$	-1.2	13	2.08	259.45	77325	61102	+Li	388.11582	388.1153
1.05	2''-O-Rhamno-sylicariside Ⅱ	$C_{33}H_{40}O_{14}$	-4.9	82	4.95	9.03	63271	17829	+Li	660.24181	660.2385
1.07	Neomangiferin	$C_{25}H_{28}O_{16}$	4.6	31	30.32	7.24	106014	27431	+Li	584.13773	584.1405
1.10	6-Aldehydo-isoophio-pogonone A	$C_{19}H_{14}O_{7}$	-2.6	10	3.33	32.95	7911	5246	+Li	354.07395	354.0730
1.10	Viscum neoside VI	$C_{24}H_{26}O_{12}$	0.1	41	1.21	560.48	274183	121351	+Li	506.14243	506.1425
1.13	Liquiritigenin-4′-O-β-D-glucopyranoside	$C_{21}H_{22}O_{9}$	0.0	18	7.95	55.89	326003	214084	+Li, +Na	418.12638	418.1264
1.16	5-Hydrox	$C_{23}H_{24}O_{11}$	-0.8	56	45.67	76.46	114489	78213	+Li, +Na	476.13186	476.1315

	y-6,4'-dimethoxy-flavone-7-O-β-D-glucopyranoside									
1.16	(2R,3R)-3,7-Dihydroxy-6-methoxyflavanone	C16H14O5	2.8	9	3.52	80.30	4609	2955	+Li	286.08412	286.0849
1.19	Mangiferitin	C13H8O6	-0.8	4	115.89	69.98	5815	4007	+NH4, +Li	260.03209	260.0319
1.21	3,4-Dihydroxy-6,7,3',4'-tetramethoxyflavone	C19H20O7	-4.3	24	6.38	287.42	109410	69665	+Li, +Na	360.12090	360.1193
1.24	Sanggenon G	C40H38O11	0.7	26	1.97	32.09	45745	10365	+H	694.24141	694.2419
1.25	Neocomplanoside	C24H24O12	0.7	30	9.89	8.39	44547	29206	+Li	504.12678	504.1271
1.28	5,6,4′-Trihydroxy flavone-7-O-β-D-galactonic acid	C22H22O11	0.9	26	3.78	468.21	133891	104203	+Li	462.11621	462.1166
1.29	Irisflorentin	C20H18O8	-3.7	12	4.11	42.16	14859	8213	+Li	386.10017	386.0987
1.31	Cyclomorusin	C25H22O6	-3.0	18	16.06	11.37	94662	56949	+Li	418.14164	418.1403
1.33	Benzopyran derivative Ⅲ	C32H28O10	-0.1	31	1.48	29.80	184633	95227	+Li	572.16825	572.1682
1.33	Prunetin-4'-glucoside	C22H22O10	-0.7	36	28.07	16.53	64378	39819	+Li	446.12130	446.1210

1.33	Isoaloeresin D	C29H32O11	-0.7	70	1.48	3900.01	1366121	856283	+Li, +Na	556.19446	556.1941
1.37	Gardenin C	C20H20O9	-3.0	9	5.76	54.02	14786	11156	+Li	404.11073	404.1095
1.39	6,7-Dihydroxy-2-(2-phenylethyl) chromone	C19H18O4	-4.4	5	5.31	139.68	13598	7540	+NH4	310.12051	310.1191
1.40	2''-O-Feruloylaloesin	C29H30O12	-3.3	45	8.97	57.11	157621	13567	+Li	570.17373	570.1718
1.45	Nelumboroside A	C27H30O16	0.7	76	1.15	28.38	199697	23240	+Na, +K	610.15338	610.1538
1.47	Odoratin-7-O-β-D-glucoside	C22H24O9	-1.6	37	7.76	9.42	107809	81550	+Li, +Na	432.14203	432.1413
1.47	Acacetin-7-O-(6''-O-acetyl)-β-D-glucopyranoside	C24H24O11	0.0	49	11.77	8.52	297088	59515	+Li, +Na	488.13186	488.1319
1.48	7-Hydroxy-5,8-dimethoxyflavone-7-O-β-D-glucoside	C23H24O10	-1.9	35	3.81	9.23	73481	47528	+Li	460.13695	460.1361
1.51	Chrysin-7-O-β-D-glucopyranoside	C21H20O9	-1.1	19	1.99	147.19	52492	33502	+Li	416.11073	416.1103
1.55	Isorhamnetin-3-O-β-D-rutinoside	C30H40O16	2.0	33	2.24	53.92	52184	7580	+Na	656.23164	656.2330

1.55	Shegans u A	C32H32 O16	-0.6	35	2.21	34.26	16369	3334	+Na	672.1690 3	672.168 6
1.56	3′-O-Methylt axifolin	C16H14 O7	4.0	6	4.94	2308.35	10192	5972	Sn	318.0739 5	318.075 7
1.61	6-Hydrox ykaemp ferol-3-O-glucosi de	C21H20 O12	1.0	14	0.90	1.19	147157	102705	+Na, +K , +Li	464.0954 8	464.096 0
1.61	Pinnatif ine I	C23H20 O10	-4.9	16	5.54	59.50	11463	7808	+H	456.1056 5	456.103 4
1.64	Kushen ol N	C26H30 O7	-0.4	18	2.17	9.15	42110	27881	+Na	454.1991 5	454.199 0
1.65	Kaempf erol-3-O-neohesp eridosid e	C27H30 O15	0.6	51	11.98	30.12	4741	3409	+Na	594.1584 7	594.158 8
1.67	Silydian in	C25H22 O10	-2.9	32	3.23	39.48	11219	6883	+Li	482.1213 0	482.119 9
1.68	(5S,6S,7 R,8S)-2-(2-Phenyle thyl)-6,7,8-trihydro xy-5,6,7,8-tetrahy dro-5-[2-(2-phenyle thyl)-7-hydrox ychrom one-6-oxy]chr omone	C34H30 O9	-2.1	50	7.59	4.15	34616	4766	+K	582.1889 8	582.187 7
1.85	Cartorm in	C27H29 NO13	4.2	57	4.00	29.84	277515	179065	+NH4	575.1638 9	575.166 4
1.86	7-O-β-D-Glucop yrano-syl-	C21H20 O11	-1.1	34	21.13	5.01	25273	16273	+Na, +K	448.1005 6	448.100 1

	kaempferol										
1.86	Ononin	C22H22O9	-0.1	34	15.35	38.85	53578	34784	+Li	430.12638	430.1263
1.87	Quercetin-3-O-α-L-arabinofuranoside	C20H18O11	-0.6	22	4.20	11.38	14878	9404	+Na	434.08491	434.0846
1.88	6-Methoxy-2-(2-phenylethyl)chromone	C18H16O3	-3.8	28	6.80	51.14	24016	9412	+NH4	280.10994	280.1088
1.89	Icariin	C33H40O15	-4.7	106	32.52	2.07	1895480	10595	+H	676.23672	676.2335
1.89	Sanggenon A	C25H24O7	-0.7	31	7.08	12.10	65599	27496	+Li	436.15220	436.1519
1.92	Kushenol V	C21H22O7	-1.4	10	5.31	15.44	28115	3132	+H	386.13655	386.1360
1.97	Isosakuranetin-7-rutinoside	C28H34O14	3.5	60	21.77	16.47	87251	22605	+H	594.19486	594.1970
1.98	3',4',5',5,7,8-Hexame-thoxy flavone	C21H22O8	0.1	20	1.42	120.49	40082	27504	+Li	402.13147	402.1315
1.98	8-C-Prenylkaempferol	C20H18O6	-0.9	29	9.54	30.61	11419	3752	+Li	354.11034	354.1100
1.99	Rhamnetin-3-O-β-D-glucopyranoside	C22H22O12	3.5	42	4.38	41.70	148963	88421	+Na, +H, +K	478.11113	478.1129
10.22	Oroxin B	C27H30O15	0.8	10	0.97	58.71	7871	4304	+H	594.15847	594.1589
15.68	Phellochinin A	C26H30O11	-3.2	47	4.08	1344.27	68519	18792	+Na	518.17881	518.1771
15.70	Neokurarinol	C27H34O7	-1.2	52	7.77	12.80	94872	36693	+H	470.23045	470.2299

2.01	Trifolin	C21H20O11	-0.2	30	4.61	1854.81	13072	8177	+Na	448.10056	448.1004
2.02	5,7,2′-Trihydroxy-flavanone-4′-O-β-D-glucoside	C21H22O11	0.7	37	0.86	18.59	72358	40753	+Na, +K, +Li	450.11621	450.1165
2.04	Aloeresin G	C29H30O10	0.0	57	11.33	128.66	447577	244616	+Li, +Na	538.18390	538.1839
2.05	Viscumneoside Ⅲ	C27H32O15	3.6	56	37.75	8.88	35642	19247	+Li	596.17412	596.1763
2.10	8,3′-Dihydroxy-7,4′-dimethoxy-isoflavone	C17H14O6	-1.4	5	1.90	13.81	6422	5395	+Na	314.07904	314.0786
2.14	Kushenol G	C25H28O8	-4.9	24	5.00	14.99	53064	30538	+H	456.17842	456.1762
2.22	Naringenin4′-O-glucopyranoside	C21H22O10	-0.2	27	3.13	15.87	20351	13821	+Na, +Li	434.12130	434.1212
2.23	5-Hydroxy-6,7-dimethoxyflavone-4'-O-β-D-glucoside	C23H24O11	1.2	33	1.47	54.93	30103	24333	+Na	476.13186	476.1324
2.25	Methylkushenol C	C26H28O7	-2.5	34	44.79	66.65	15848	11145	+Li	452.18350	452.1824
2.30	(-)-Epiafzelechin-3-O-(6"-O-acetyl)-β-D-allosepy	C23H26O11	0.2	30	0.64	16.78	18780	13807	+Na	478.14751	478.1476

	ranoside										
2.31	3'-Hydroxypuerarin	C21H20O11	-0.8	13	1.05	16.13	18576	12742	+Na, +H	448.10056	448.1002
2.33	Ledebouriellol	C20H22O7	-1.0	16	4.69	37.14	18016	12770	+Na	374.13655	374.1361
2.33	Sagittatoside B	C32H38O14	-4.7	57	4.14	126.14	245703	6573	+H	646.22616	646.2231
2.40	Ginkgetin	C32H22O10	1.3	15	16.73	22.28	27328	19437	+Li	566.12130	566.1220
2.46	Tetuin	C21H20O10	-2.1	7	3.14	98.20	2072	2072	+Na	432.10565	432.1047
2.46	Cnidimol E	C15H16O6	1.9	8	1.92	22.07	4962	3948	+Na	292.09469	292.0953
2.58	Noranhyoicaritin	C21H22O6	1.5	32	4.98	35.55	60813	5664	+Li, +H	370.14164	370.1422
2.62	Cryptomerin B	C32H22O10	-4.1	10	18.35	43.05	24126	16591	+Li	566.12130	566.1189
2.65	Bavachinin	C21H22O4	4.2	20	3.96	81.79	46719	19635	+Li	338.15181	338.1533
2.74	Morusin hydroperoxide	C25H24O8	-0.2	21	1.34	115.73	38215	26070	+Li, +Na	452.14712	452.1470
2.81	Tangeritin	C20H20O7	-0.6	3	3.32	37.60	65532	46159	+Li, +Na	372.12090	372.1207
2.89	Leachianone A	C26H30O6	-1.4	45	12.61	32.33	56311	9108	+K	438.20424	438.2036
2.90	2′-Hydroxy-4′,6′-dimethoxydihydrochalcone	C17H18O4	-1.4	15	2.56	12.74	10490	6107	+H	286.12051	286.1201
2.90	Kaempferol-3-gentiobioside	C27H30O16	4.9	56	5.44	36.53	6254	4420	+H	610.15338	610.1564
3.06	3',4',7-Tribenzylsappanol	C37H34O6	4.7	11	4.90	24.82	60279	39633	+H	574.23554	574.2383
3.28	Liquiritin apioside	C26H30O13	3.6	24	3.34	38.70	124875	69508	+Na, +K, +NH4	550.16864	550.1707

3.28	5′-Methoxy-bilobetin	C32H22O11	0.4	9	3.54	24.35	18701	12926	+Li	582.11621	582.1164
3.36	Paeonin	C28H33ClO16	-2.0	15	6.42	16.29	97426	2916	+H	660.14571	660.1444
3.36	Pectolinarin	C29H34O15	1.7	41	2.32	31.01	96378	15282	+Na, +K	622.18977	622.1909
3.37	Morusinol	C25H26O7	-0.3	32	7.41	11.27	57090	18567	+Li, +Na	438.16785	438.1677
3.37	Crenatoside	C30H28O11	-1.1	33	1.57	68.97	30006	14159	+Li, +Na	564.16316	564.1626
3.67	3-Hydroxynaringenin	C15H12O6	-1.6	10	2.02	43.21	10227	5401	+Na, +H	288.06339	288.0629
3.76	Kushenol Q	C26H32O7	1.6	30	8.49	23.22	14240	6631	+Na	456.21480	456.2155
3.98	Sanggenon I	C25H26O6	-4.2	15	5.41	100.74	40705	23323	+Li	422.17294	422.1711
4.11	Cnidimol D	C15H16O6	2.4	10	2.39	19.28	7506	6081	+Na	292.09469	292.0955
4.13	Kuwanon M	C50H48O12	4.8	19	4.67	38.49	22323	9773	+Na, +K	840.31458	840.3188
4.17	(-)-Epicatechin-pentaacetate	C25H24O11	-1.9	9	13.87	124.40	11035	6414	+Li	500.13186	500.1309
4.26	Lupinifolin	C25H26O5	-2.3	5	33.44	31.90	64853	37345	+Li, +Na	406.17802	406.1771
4.32	Ophiopogonanone B	C18H18O5	-3.4	6	3.37	15.27	15210	6338	+Na, +H	314.11542	314.1143
4.55	2′,7-Dihydroxy-4′,5′-dimethoxyisoflavone	C17H14O6	-1.3	4	1.72	17.30	8134	6576	+H	314.07904	314.0786
4.89	2′-O-Methylisoliquiritigenin	C16H14O4	-1.5	3	3.70	63.45	18702	12796	+H	270.08921	270.0888
5.60	6,8-Dihydroxy-2-[2-(4'-hydrox	C17H14O5	-1.1	6	1.29	20.09	37806	31674	+H	298.08412	298.0838

	yphenyl)ethyl] chromone									
5.80	Norcimifugin	C15H16O6	4.8	13	4.70	33.03	3605	2727	+Na	292.09469	292.0962
5.81	4′,7-Dimethyltectori-genin	C18H16O6	-0.8	12	1.73	19.56	32349	23213	+H, +Na	328.09469	328.0944
6.01	(3R,4S)-3,4-Dihydroxy-3-(3′,4′-dimethoxybenzyl)-7-methoxy-chroman	C19H22O6	-3.4	21	32.34	14.63	27858	16869	+Li	346.14164	346.1404
6.05	6-Methoxy-2-[2-3'-methoxy-4'-hydroxy-phenyl)ethyl]chromone	C19H18O5	-1.2	17	2.25	33.71	139719	58614	+H, +Na, +Li	326.11542	326.1150
6.16	7-Hydroxy-5,3',4'-trimethoxy flavone	C18H16O6	-0.6	18	1.00	5.53	84396	62768	+H, +Na	328.09469	328.0945
6.25	Flavonol	C15H10O3	0.2	2	3.72	236.73	77981	67082	+Li, +Na	238.06299	238.0630
6.37	Ophiopogonanone A	C18H16O6	-3.7	21	3.53	42.48	6798	5040	+Na	328.09469	328.0934
6.58	Methylophiopogonanone B	C19H20O5	-2.2	15	2.05	14.76	83994	33142	+H, +Na	328.13107	328.1304
6.86	5,7-Dihydro	C9H6O4	3.8	1	4.65	10.07	10112	9285	+NH4	178.02661	178.0274

	xychromone										
7.44	Rhodanthenone D	C14H10O7	-4.2	0	4.74	312.38	13700	13700	+K	290.04265	290.0413
7.94	5,7,4′-Trimethoxy flavone	C18H16O5	-1.6	26	1.84	9.87	237078	186903	+H, -e	312.09977	312.0993
8.04	3'-Deoxysappanone B	C18H18O5	-0.8	3	1.03	1.74	144471	88263	+Na, +K, +Li	314.11542	314.1152
8.35	5,7,3',4'-Tetramethoxyflavone	C19H18O6	-0.5	40	0.60	1.49	332210	239816	+H, +Li	342.11034	342.1102
8.68	Mahuannin J	C30H20O11	-2.1	1	2.56	26.39	2170	1486	+NH4	556.10056	556.0994
9.10	Viscumneoside IV	C29H32O16	-5.0	33	4.77	16.87	12429	5153	+K	636.16903	636.1657
